# Structure, morphology, and magnetic properties of Fe nanoparticles deposited onto single-crystalline surfaces

**DOI:** 10.3762/bjnano.2.6

**Published:** 2011-01-21

**Authors:** Armin Kleibert, Wolfgang Rosellen, Mathias Getzlaff, Joachim Bansmann

**Affiliations:** 1 Swiss Light Source, Paul Scherrer Institut, 5232 Villigen, Switzerland; 2 Institut für Angewandte Physik, Universität Düsseldorf, 40225 Düsseldorf, Germany; 3 Institut für Oberflächenchemie und Katalyse, Universität Ulm, 89081 Ulm, Germany

**Keywords:** epitaxy, iron, magnetic nanoparticles, Ni(111), RHEED, spontaneous self-alignment, STM, W(110), XMCD

## Abstract

**Background:** Magnetic nanostructures and nanoparticles often show novel magnetic phenomena not known from the respective bulk materials. In the past, several methods to prepare such structures have been developed – ranging from wet chemistry-based to physical-based methods such as self-organization or cluster growth. The preparation method has a significant influence on the resulting properties of the generated nanostructures. Taking chemical approaches, this influence may arise from the chemical environment, reaction kinetics and the preparation route. Taking physical approaches, the thermodynamics and the kinetics of the growth mode or – when depositing preformed clusters/nanoparticles on a surface – the landing kinetics and subsequent relaxation processes have a strong impact and thus need to be considered when attempting to control magnetic and structural properties of supported clusters or nanoparticles.

**Results:** In this contribution we focus on mass-filtered Fe nanoparticles in a size range from 4 nm to 10 nm that are generated in a cluster source and subsequently deposited onto two single crystalline substrates: fcc Ni(111)/W(110) and bcc W(110). We use a combined approach of X-ray magnetic circular dichroism (XMCD), reflection high energy electron diffraction (RHEED) and scanning tunneling microscopy (STM) to shed light on the complex and size-dependent relation between magnetic properties, crystallographic structure, orientation and morphology. In particular XMCD reveals that Fe particles on Ni(111)/W(110) have a significantly lower (higher) magnetic spin (orbital) moment compared to bulk iron. The reduced spin moments are attributed to the random particle orientation being confirmed by RHEED together with a competition of magnetic exchange energy at the interface and magnetic anisotropy energy in the particles. The RHEED data also show that the Fe particles on W(110) – despite of the large lattice mismatch between iron and tungsten – are not strained. Thus, strain is most likely not the origin of the enhanced orbital moments as supposed before. Moreover, RHEED uncovers the existence of a spontaneous process for epitaxial alignment of particles below a critical size of about 4 nm. STM basically confirms the shape conservation of the larger particles but shows first indications for an unexpected reshaping occurring at the onset of self-alignment.

**Conclusion:** The magnetic and structural properties of nanoparticles are strongly affected by the deposition kinetics even when soft landing conditions are provided. The orientation of the deposited particles and thus their interface with the substrate strongly depend on the particle size with consequences regarding particularly the magnetic behavior. Spontaneous and epitaxial self-alignment can occur below a certain critical size. This may enable the obtainment of samples with controlled, uniform interfaces and crystallographic orientations even in a random deposition process. However, such a reorientation process might be accompanied by a complex reshaping of the particles.

## Introduction

Ferromagnetic clusters and nanoparticles have gained huge interest due to their interesting fundamental properties as well as their possible applications in data storage media, chemistry, biotechnology and medicine [1–4]. First, Stern–Gerlach measurements proved that ferromagnetic particles may exhibit enhanced and strongly size-dependent magnetic moments [5]; and even non-magnetic materials can show ferromagnetism at the nanoscale [6]. In these systems, the enhanced magnetism in clusters is basically ascribed to the high surface-to-volume ratio. Similarly to magnetic thin films or surfaces, the reduced coordination at the cluster surface leads to significantly higher magnetic spin moments compared to the respective bulk materials. In *3d* transition metals, the magnetic spin moment is large compared to the orbital moment. The orbital moment is usually strongly quenched in bulk materials in comparison to single atoms, and thus, the spin moment is mainly responsible for the total magnetization of the material. However, the orbital moment is an important microscopic quantity in magnetism since it is strongly related to the magneto-crystalline anisotropy energy which, in turn, determines many macroscopic properties of magnetic samples [7–8]. Moreover, the orbital moments are much higher at surfaces or in clusters than in the bulk [9–12]. As a consequence, the orbital contribution to the total magnetization – although small in magnitude – is of fundamental interest, especially in low dimensional systems (for an overview see, e.g., [1,13–15]). For any application the particles have to be supported by or be embedded into a suitable medium. Many studies showed that the cluster–substrate interaction has an tremendous impact on the resulting magnetic properties. The physical origin of such a behavior may be caused, e.g., by strain effects resulting from the interface or a (often anisotropic) compression of the lattice (such as phase transitions from bcc to bct), a phenomenon well-known from ultrathin films on single crystalline surfaces [16]. In special cases, the magnetic anisotropy energy in the nanoparticle may be orders of magnitudes larger than in bulk-like materials. An example for such a case is given by FeCo alloy nanoparticles, where extremely high magnetic anisotropy energies have first been predicted [17] and later on found experimentally in thin films and nanoparticles [18–20].

For technical applications of nanoparticles, homogeneous size distribution is of great importance to guarantee comparable physical properties in an ensemble of particles. Chemistry-based routines [21–23], reaching both a homogeneous particle size and a well-ordered arrangement, are appropriate methods for large-area applications. However, besides homogeneous size-distribution, uniformity in the particle orientation might also be desired. This is particularly important in magnetic devices where well-defined magnetization axes and switching fields are required to store or to process information. Since it is known that even mono-dispersed particles can show significant variations in their magnetic anisotropy energies and in the orientation of their magnetization axes [24], strategies to achieve uniformity in these properties are highly demanding. Physically based nanoparticle preparation techniques are less favorable for large-area applications, but they often provide better control over interfaces and purity and are thus more appropriate for fundamental research and characterization of the magnetic properties. Particularly, they avoid the additional complexity given by the presence of solvents and ligands used in wet-chemical techniques. Thus, to unveil the processes that happen when pure nanoparticles come in contact with a well-defined substrate, we have investigated mass-filtered Fe nanoparticles being deposited from the gas phase onto single-crystalline surfaces under soft-landing conditions. In particular, we combine in situ (i) XMCD to determine their magnetic spin and orbital moments, (ii) RHEED to get access to their crystallographic structure and orientation and (iii) STM to observe their real space morphology on the substrate. The goal of the manuscript is to correlate the structure and the morphology of deposited iron nanoparticles with their magnetic properties.

## Experimental

Experiments on exposed mass-filtered Fe nanoparticles on (ferromagnetic) supports require in situ cluster deposition as well as surface sensitive analysis techniques performed under ultrahigh vacuum conditions. To motivate the need of our combined approach, we first introduce the arc cluster ion source (ACIS) and its deposition characteristics before presenting the magnetic and structural data obtained by XMCD, RHEED and STM.

### Cluster generation and deposition by means of ACIS

**Figure 1:**
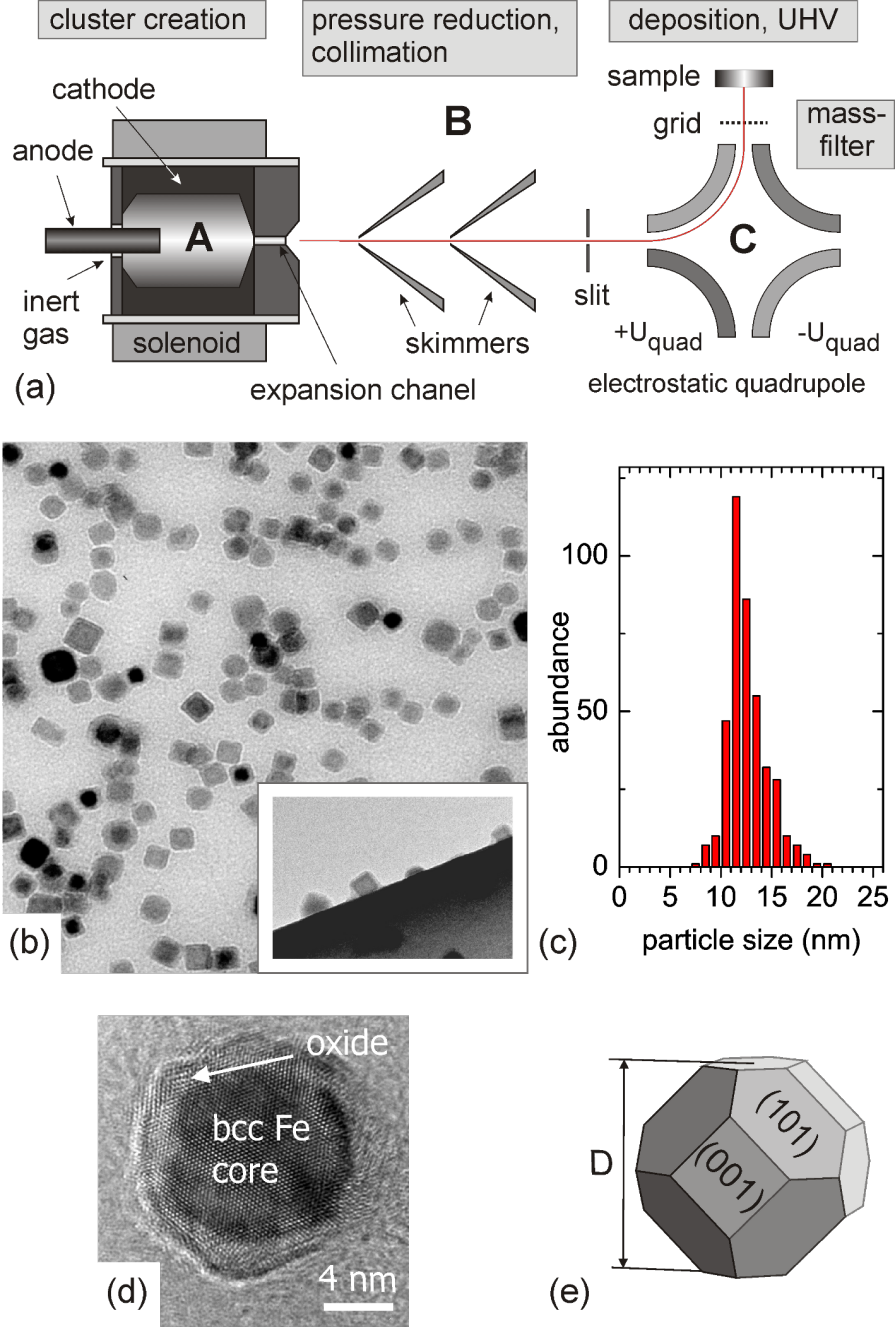
(a) Schematic drawing of the ACIS. (b) TEM image of a typical nanoparticle deposit on a carbon coated copper grid. The side view in the inset reveals compact three-dimensional shapes. (c) Size distribution of Fe particle sizes from (b) including the oxide shell. (d) High resolution TEM image of a single particle showing a 2 nm thick oxide shell surrounding the crystalline Fe core. (e) Schematics of the equilibrium shape of pure crystalline Fe particles as expected from a Wulff construction in the present particle size range [32].

Fe nanoparticles are generated in the gas phase using a continuously working ACIS for nanoparticle deposition experiments (Figure 1). The design of this source is based on the experience with the pulsed arc cluster ion source (PACIS) in the group of Meiwes-Broer (University of Rostock, Germany) [25]. There, the PACIS is mainly used to produce small size-selected clusters for gas phase experiments in combination with pulsed lasers or other pulsed light sources [26]. For the present experiments the PACIS design has been modified to allow a high and continuous flux of mass-filtered nanoparticles (size regime: 4 nm to 25 nm) with a moderate size distribution in surface science experiments [27–28]. The resulting ACIS is ultrahigh vacuum compatible, small in size to allow easy transportation and can be flexibly attached to different experimental stations, e.g., laboratory-based STM experiments, different end stations at synchrotron light sources such as BESSY (Berlin, Germany) and more recently, the Elmitech PEEM at the SIM beamline of the Swiss Light Source of the Paul Scherrer Institute (Villigen, Switzerland) [29].

The ACIS consists of three different stages as shown in Figure 1a: (A) the cluster aggregation part based on a hollow cathode made from the target material (here: Fe with a purity of higher than 99%), (B) a dual pumping stage with an oil-free roots pump (250 m^3^·h^−1^) and a turbo molecular pump (250 l·s^−1^) to reduce the huge amount of noble gas (Ar and He) required for the erosion process and (C) a mass-filtering unit based on an electrostatic quadrupole. The cluster material is eroded from the hollow cathode in the presence of the noble gas (at a pressure around 20–40 mbar) using an arc discharge. Small aggregates of this material are kinetically accelerated by collisions in the nozzle and in a weak supersonic expansion to almost the velocity of the seeding gas. The composition of the carrier gas (Ar and He) is controlled by two individual mass flow controllers. After pumping off most of the noble gas, the Fe nanoparticles enter the electrostatic quadrupole deflector which acts as a mass-filtering unit. Different from other gas aggregation sources [30], about 50% of the particles are positively and negatively charged (mostly single charged) and are thus deflected in the electric field of the electrostatic quadrupole. Due to their nearly constant velocity after the expansion process in the hollow cathode, the kinetic energy of the particles is directly related to their mass allowing a separation by using an electrostatic energy dispersive element. The particle deposition finally takes place in a UHV preparation chamber at a residual (noble gas) pressure of about 1 × 10^−6^ mbar during operation of the source. More recently, a set of aerodynamic lenses has been added to increase the available particle size range and to improve the cluster flux [31].

The size distribution and structure of the Fe particles in the mass-selected cluster beam is deduced from ex situ transmission electron microscopy (TEM). A typical (air exposed) nanoparticle deposit on a carbon coated copper grid is given in Figure 1b. The side view in the inset shows compact particles with mostly cubic shapes due to the partial oxidation. Electron diffraction reveals the presence of metallic bcc Fe and Fe oxides as, e.g., Fe_3_O_4_ [27]. A size distribution of the oxidized Fe particles displayed in Figure 1b is given in Figure 1c and yields *D*_oxi_ = (12.0 ± 1.4) nm. To obtain the actual mean size of the pure Fe nanoparticles, the distribution in Figure 1c needs to be corrected for the effect of partial oxidation. High resolution TEM of the exposed particles reveals metallic cores surrounded by an oxidic shell with a thickness of about 2 nm as shown in Figure 1d. The respective size correction then leads to *D* = (9.6 ± 1.5) nm for the pure particles before air exposure. High resolution TEM images also reveal that the metallic cores are single crystalline with bcc structure in most of the particles. Similar observations are reported for Fe particle sizes down to 4 nm [33–34]. We may note that at smaller sizes transitions to other structures may occur and thus experimental characterization is always important [35–37].

Energy considerations show that single crystalline bcc Fe nanoparticles in the size range from 4 to 10 nm have an equilibrium shape given by six (001) and twelve (110) surface facets according to a Wulff construction [32–33]. A schematic drawing of such a particle with a diameter *D* is shown in Figure 1e. This shape has also been found experimentally in pure Fe particles generated by the ACIS source [38]. The low kinetic energy per atom (usually less than 0.1 eV per atom) of the nanoparticles allows soft-landing deposition experiments well below the fragmentation threshold [39]. Accordingly, recent STM studies revealed that the shape of larger particles (*D* > 4 nm) is only weakly affected when being deposited onto single crystalline substrates, i.e., the height of supported particles as measured with STM corresponds well with the size being determined by TEM [28,40–41]. This is a particular strength of soft-landing techniques. Therefore, they also allow to obtain particle deposits far away from thermal equilibrium which otherwise are hardly accessible.

These advantages make particle deposition techniques very attractive for fundamental research on size-dependent phenomena. However, even under soft-landing conditions, the deposition process cannot be neglected in experiments. Indeed, as we will show, the deposition kinetics may have a crucial impact on the resulting particle properties: First, the random nature of the deposition technique is expected to lead to a large variety of orientations of the particles. For instance, the Fe nanoparticles studied here may finally rest with their (110) or their (001) facets on the substrate with arbitrary azimuthal orientation (Figure 1e). The different contact interfaces may have a strong impact on the particle properties, e.g., due to hybridization effects, interface-induced strain or magnetic anisotropy contributions. The influence of the interface is size-dependent and increases with decreasing particle size and may even dominate over intrinsically size-dependent properties. Second, kinetic energy and interface energy are released upon landing on the surface. The resulting heat may rapidly anneal the particles before the thermal energy is dissipated into the substrate. Depending on the available total energy and the size of the particles, the latter may realign or even reshape on the surface with respective consequences for their resulting properties.

## Results and Discussion

In previous works it was found that magnetic nanoparticles show surprisingly strong variations in their properties – such as the magnetic anisotropy energies or microscopic spin and orbital contributions to the total magnetization – when being in contact with different substrates or embedded into different matrices [20,38,42–44]. Here, we focus on Fe nanoparticles being deposited onto different magnetic and non-magnetic single crystalline surfaces. Single crystalline substrates were chosen to provide well-defined and atomically flat substrates. Magnetic substrates are used to magnetize the particles along a well-defined direction by employing the strong exchange interaction at the interface to suppress possible superparamagnetic fluctuations of the particle magnetization [8]. Previous measurements on Fe nanoparticles deposited onto hcp Co(0001)/W(110) revealed bulk-like magnetic spin moments, but surprisingly large orbital moments being twice as large when compared to the respective bulk value [38]. To study the influence of the substrate on these properties, Fe nanoparticles (NPs) have now been investigated on fcc Ni(111)/W(110).

**Figure 2:**
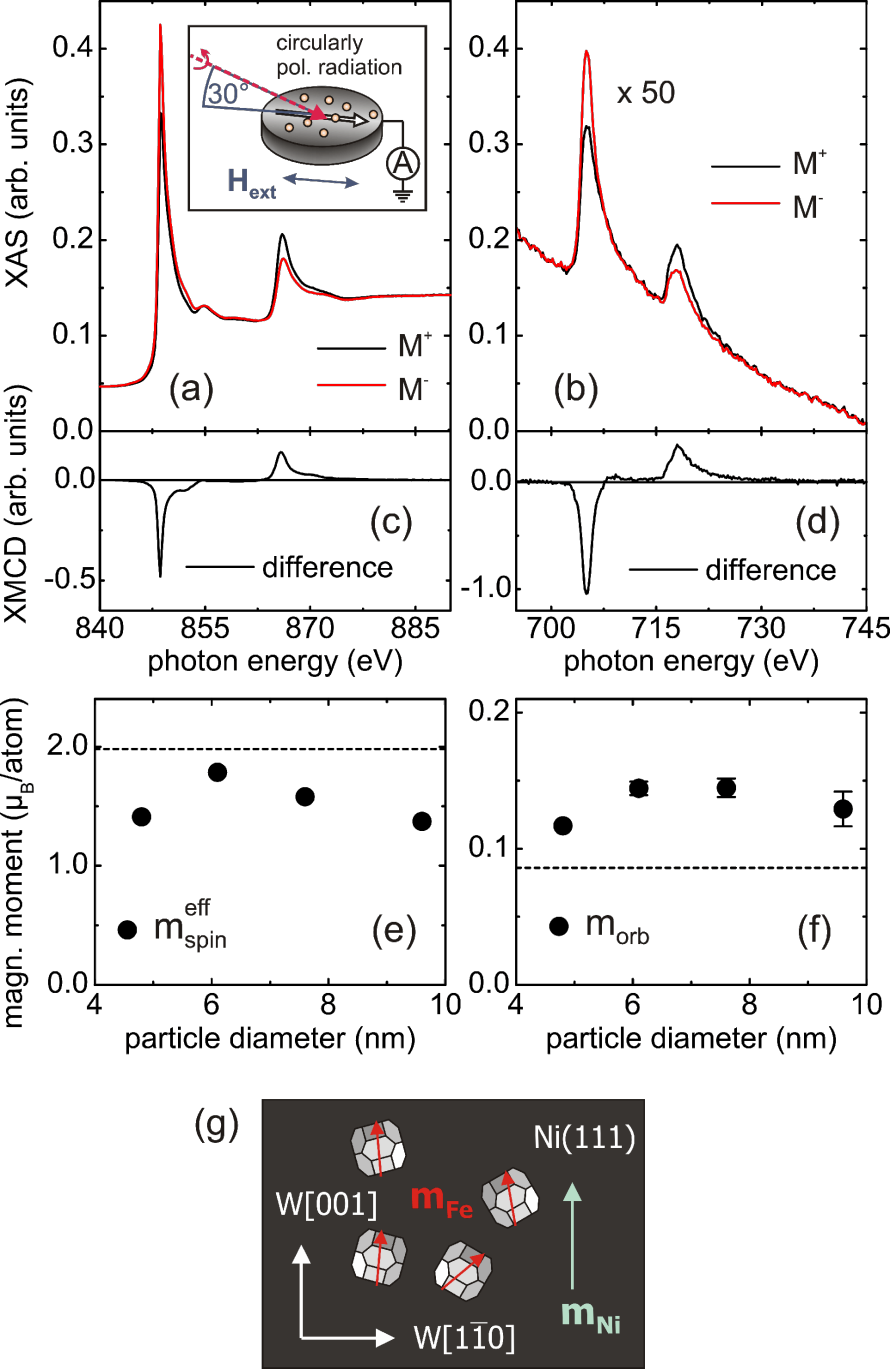
(a) X-ray absorption spectra of the Ni(111) substrate. Inset: Schematics of the experiment. (b) XAS spectra of the Fe NPs with a diameter of (7.6 ± 1.5) nm. (c),(d): Ni- and Fe-related XMCD spectra. Lower part: Size-dependent magnetic spin moments (e) and magnetic orbital moments (f) of the Fe NPs. (g) Sketch of the magnetization directions (*m*_Ni_, *m*_Fe_) of the sample as discussed further below.

The W(110) substrates were obtained by cycles of heating in oxygen atmosphere as described in the literature [45]. The Ni films with a thickness of about 15 ML were grown by thermal evaporation at a rate of 0.1 atomic monolayers per minute. To obtain a flat and relaxed surface, the films were thermally annealed at 320 K. The clean W(110) surface and the structural quality of the films were checked by means of low energy electron diffraction (LEED). Details regarding the growth and magnetic properties of Ni films on W(110) can be found in the literature [46–48]. Subsequently to film preparation, mass-filtered Fe nanoparticles were deposited from the ACIS cluster source. Figure 2a and Figure 2b show X-ray absorption spectra in the vicinity of the *L*_2_ and *L*_3_ edges of both, the Ni(111) substrate and Fe nanoparticles with *D* = (7.6 ± 1.5) nm, respectively. The data were recorded with circularly polarized synchrotron radiation provided by the helical undulator beamline UE46-PGM1 at the electron storage ring BESSY (Berlin).

The experimental setup is shown in the inset of Figure 2a, the X-rays impinge at an angle of 30° at the sample. The substrate is oriented with its easy magnetic axis, i.e., the W[001]-direction, parallel to the plane of incidence. The data were obtained by recording the total electron yield at each photon energy and by switching the Ni film magnetization with a short external magnetic field pulse at each data point (a current of ≈100 A through two coils, 180 windings, magnetic field ≈1700 G). The photon helicity was kept fixed. Note that the nanoparticle data in Figure 2b are scaled by a factor of 50. The low magnitude of the Fe signal relative to that of the Ni spectra reflects a well diluted deposit with about 200 particles per μm^2^ on the surface [38]. At this density, interactions between the particles can be neglected.

Figure 2c and Figure 2d show magnetic dichroism spectra (given by the difference of the XA spectra with opposite magnetization directions, M^+^ and M^−^, respectively) for both components, the Ni films as well as the Fe NPs. The identical sign in both XMCD spectra reveals a ferromagnetic (parallel) coupling of the particles to the substrate magnetization. This behavior is expected due to the strong exchange coupling of the interface. Applying the XMCD sum rules as shown in [49–50] reveals the magnetic moments of the substrate and the nanoparticles, respectively. For the Ni films bulk-like moments are found, thus indicating fully saturated magnetization [51]. In the case of the Fe particles self-saturation effects – which lead to a significant underestimation of the magnetic spin and more importantly of the magnetic orbital moments – have to be corrected. These effects are well-known for thin films [52] and their description has recently been extended to supported nanoparticles [53]. After further corrections regarding the incomplete degree of circular polarization, the angle of 30° between the photon propagation vector and the sample magnetization, the effective magnetic spin and orbital moments were obtained as displayed in Figure 2e and Figure 2f (details of the data analysis are described in [38]). The spin moments in Figure 2e vary slightly with the particle size but are always well below the corresponding Fe bulk value of 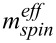 = 1.98 μ*_B_* [49] (dashed line). In contrast, the magnetic orbital moments presented in Figure 2f are, in all cases, well above the corresponding bulk value of *m*_orb_ = 0.085 μ*_B_* [49] (dashed line).

The magnitude of the orbital moments is similar to our previous findings on Fe nanoparticles in contact with Co(0001)/W(110) [38]. A detailed analysis showed that the observed moments are not explained by the well-known enhancement of the orbital magnetic moments at bulk surfaces or, respectively, nanoparticle surfaces (e.g., [10] and references therein). Instead, the data suggest that the orbital moments are also altered in the particle volume. From the literature it is known that the magnetic moments in iron are highly sensitive to the actual lattice symmetry [54–55]. In [38], we therefore assumed that surface and interface related strain in the nanoparticles as, e.g., observed in [56] could be the origin of such enhanced orbital moments. Similarly, the reduced magnetic spin moments in Figure 2e could be due to, e.g., tetragonal lattice distortions. Thus, to shed more light on these findings, it is essential to directly study the structure of the particles upon deposition onto a single crystalline substrate.

**Figure 3:**
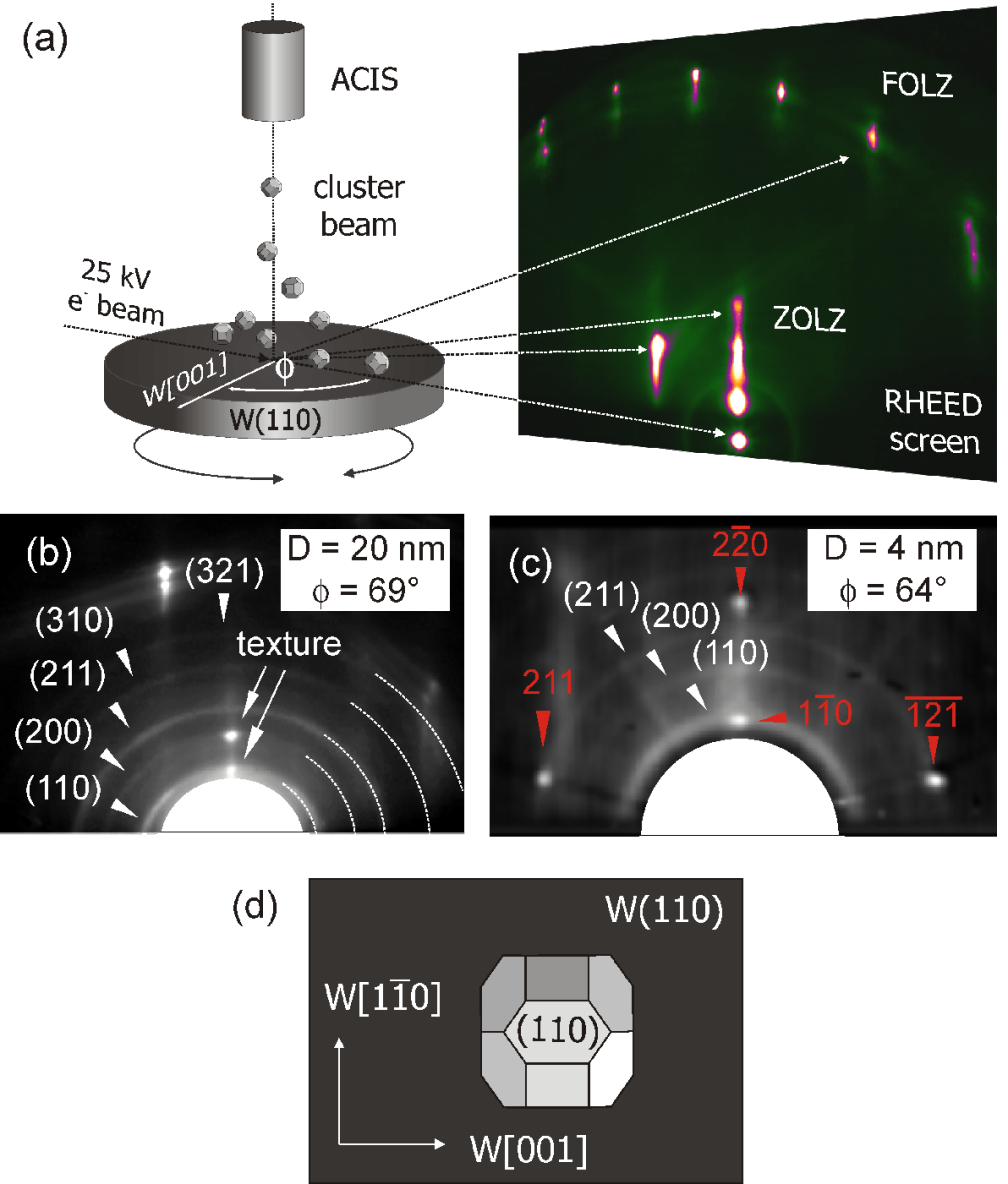
(a) Schematics of the in situ RHEED setup [58] (b) and (c): RHEED diffraction patterns from (b) larger (20 nm) and small (c) particles (4 nm). (d) Schematic drawing of uniformly oriented Fe particles on W(110).

To assess the crystallographic structure and orientation of supported particles in situ, RHEED [57] was used. A schematics of the experimental setup is shown in Figure 3a, details can be found in [58]. The experiments were performed on Fe nanoparticles upon deposition onto the bare W(110) surface. The data in Figure 3a, Figure 3b and Figure 3c are part of a recent study published in [59]. The system Fe/W(110) is particularly interesting for studying substrate-induced strain effects in deposited nanoparticles due to the large lattice misfit of 9.5% and the well-known strain relaxation in thin Fe films grown on W(110). The latter gives rise to a complex interplay between structure and magnetic properties [60–61]. The grazing incidence and the high cross section of electrons with matter make RHEED ideally suited for nanoparticle experiments, even for highly diluted samples. Moreover, this method probes both, the Fe nanoparticles and the W(110) substrate, simultaneously. Thus, one can study the relative orientation of the particles with respect to the lattice of the substrates. In addition, the substrate serves as a well-defined reference for studying quantitatively possible strains in the particles.

To illustrate the method Figure 3a shows a large diffraction pattern of the bare W(110) substrate. The characteristic streaks aligned on two half circles around the central beam correspond to the zeroth and first order Laue zone (ZOLZ and FOLZ). Here, these W(110) streaks serve as a reference for the orientation of the sample and for the analysis of the diffraction pattern of the Fe nanoparticles. Deposition of mass-filtered Fe particles with a size of *D* = 20 nm results in the appearance of diffraction rings as shown in Figure 3b. This pattern – being similar to common powder diffraction data – is independent of the azimuthal sample orientation Φ and thus indicates a random orientation of the nanoparticles on the substrate. It is found for particles with sizes ranging from 25 nm down to about 4 nm. Comparing the Fe induced rings with the well-defined positions of the W(110) streaks reveals a bulk-like Fe lattice constant in the nanoparticles. Thus, despite of the large lattice mismatch with the substrate, the particles possess a bulk-like Fe lattice. A texture in the (200) and (110) rings (marked by arrows in Figure 3b) also shows that the particles preferentially rest on their (001) and (110) surface facets, however, without any preferential azimuthal orientation.

These data allow a major conclusion for the interpretation of the magnetic moments presented in Figure 2e and Figure 2f: Strain in the particles can largely be excluded as a possible origin of altered magnetic spin and orbital moments. More precisely, we may note that our size-dependent RHEED data indicate that strain might still be present in the first few layers from the interface [59], analogously to the findings in closed Fe films on W(110), where strain relaxation takes place within the first four layers [62]. However, a 6 nm particle (as shown in Figure 1e) consists of about several tens of atomic (001) or (110) layers, respectively. Thus, the major particle volume can be considered as relaxed; strain does not contribute to the altered magnetic spin and orbital moments. Before we discuss other alternative explanations for the observed magnetic moments, we may focus on another related interesting and important phenomenon that may occur when depositing nanoparticles onto single crystalline substrates.

At a particle size of 4 nm, additional angular dependent spot patterns (indicated by arrows) occur in RHEED as shown in Figure 3c [59]. Analyzing this pattern reveals the onset of a spontaneous epitaxial alignment. In particular, we observe a parallel alignment of the (110) planes and the [001] directions of Fe and W, which is well-known from Fe films grown thermally on W(110). The remaining ring pattern in Figure 3c shows that there are still some randomly oriented particles. However, below 4 nm full alignment of the Fe particles is found. Together with the above discussed Wulff shape of the particles, these findings suggest that an ensemble of particles with uniform shape and orientation on the substrate has formed (as shown in Figure 3d). However, landing and relaxation kinetics during (and after) the impact of the particles on the surface may potentially lead to significant deviations from this simple picture and thus require additional attention.

**Figure 4:**
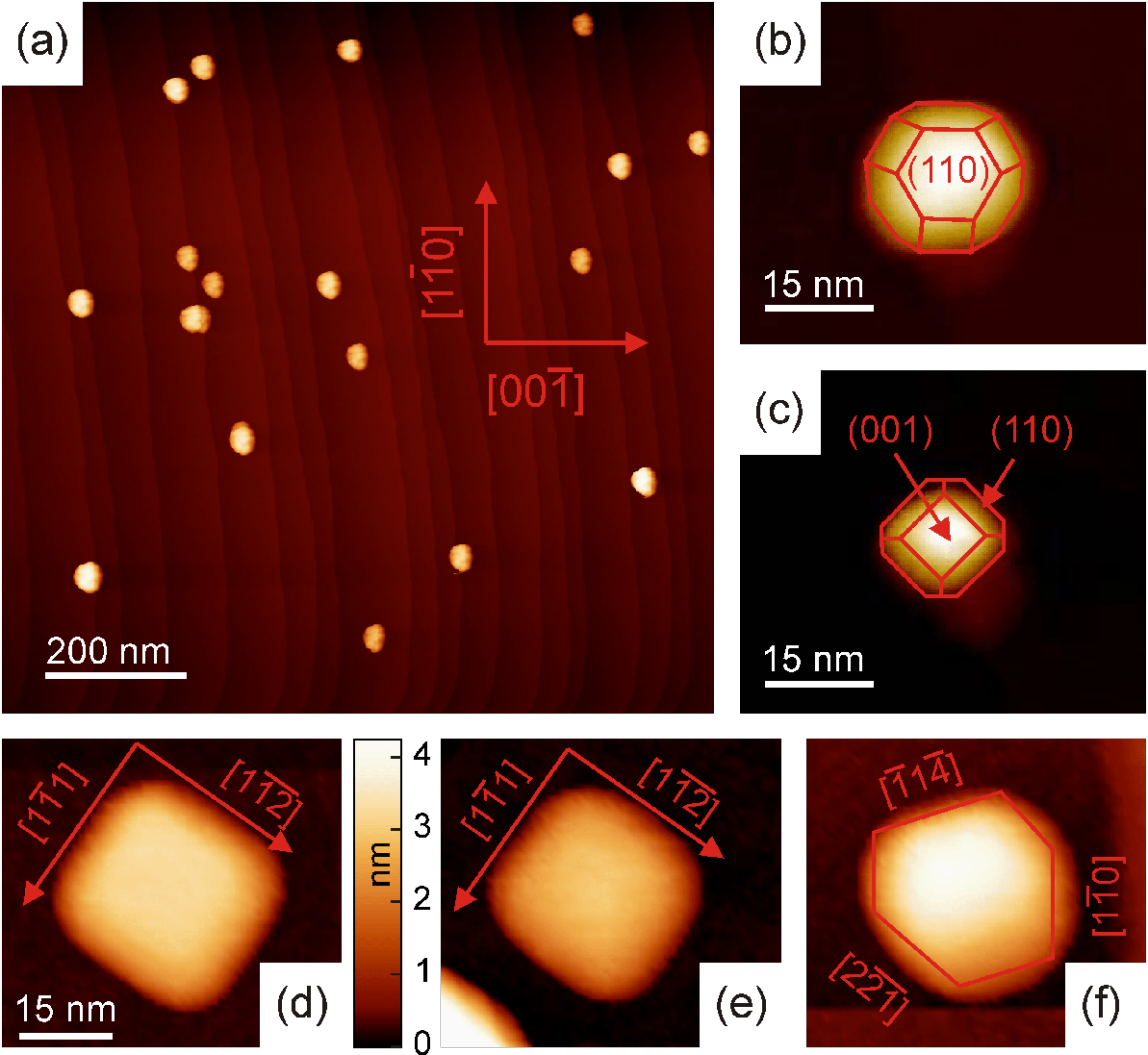
(a) STM image of mass-filtered Fe nanoparticles (mean diameter of 7 nm) deposited onto W(110). Tunneling parameters: *U* = 1.0 V, *I* = 0.5 nA. (b) and (c): Tip deconvolution of the STM images reveals surface facets consistent with the random orientation as found by means of RHEED. (d)–(f): At smaller sizes (*D* < 4 nm) some particles show edges along different crystallographic directions of the substrate. These directions do not correspond to the simple model discussed earlier (Figure 3d).

To shed more light on the deposition kinetics we have performed first STM investigations on the morphology of Fe NPs on W(110). The overview image in Figure 4a shows randomly distributed nanoparticles with a diameter of about 7 nm as deposited from the ACIS source. Analogously to earlier findings, the height measured in STM corresponds well to the respective diameter measured by TEM. The apparent lateral particle dimensions of about 50 nm in Figure 4a are due to tip convolution effects in the STM which become important when the particle size is comparable or larger than the tip diameter [63]. The tip convolution in general also superimposes details of the particle shape as, e.g., surface facets. Numerical deconvolution of the STM images has been shown to provide a tool to reconstruct features of the nanoparticle morphology [64]. Figure 4b and Figure 4c show such reconstructed images of two particles. The images indeed show indications for particle shapes according to the Wulff construction (see the schematics in the figures). Moreover, the particles in Figure 4b and Figure 4c may rest on their (001) and (110) facets with arbitrary azimuthal orientations as expected from the RHEED data for particles of this size.

When depositing smaller particles with *D* = 4 nm, we observe structural features at about 20% of the particles even without the need for numerical deconvolution as shown in Figure 4d, Figure 4e and Figure 4f. The remaining particles appear rounder or show stronger irregular tip features. The particles in Figure 4d, Figure 4e and Figure 4f show edges along certain crystallographic directions of the W(110) substrate as denoted in the figures. Thus, we attribute these more regularly shaped particles to the onset of spontaneous alignment being observed by RHEED. However, the facets seen in Figure 4d, Figure 4e and Figure 4f do not correspond to the simple model derived from the RHEED data (Figure 3d). In fact the Wullf shape model with only (001) and (110) surface facets suggests sharp edges along the {111}, {001}, and {110} directions of the substrate. Thus, the data in Figure 4d, Figure 4e and Figure 4f hint at the presence of higher index surface facets which are energetically less favorable. We therefore assume that the observed shape reflects a state far from thermal equilibrium forming due to the complex landing kinetics of the smaller particles which are connected with the spontaneous reorientation process.

Recent molecular-dynamics (MD) simulations give microscopic insight into the processes in nanoparticle deposition experiments [65]. In particular, it turns out that upon impact the particles are temporarily disordered. The subsequent recrystallization happens on a ps time scale and may result in partial or full epitaxy of the particles. Thereby, the alignment with the substrate is achieved by a thermally activated ejection of dislocations which form upon deposition. The final state of the supported particles depends on their size and the available kinetic and interface energy. Once the energy is dissipated the particles remain trapped in their respective state until further relaxation processes are activated at the present sample temperature. Our room temperature experiments show stable particle properties over periods of several hours. Thus, we conclude that the kinetic barriers for further relaxation are relatively high, and dislocations are effectively trapped in those particles which are not aligned with the substrate. Note that dislocations locally reduce the symmetry in the crystal lattice and, thus, the MD simulations together with the random orientation found for larger particles (*D* > 4 nm) may provide an alternative explanation for the strongly enlarged orbital moments presented in Figure 2f. Namely, trapped dislocations might induce enhanced magnetic orbital moments in deposited nanoparticles.

The reduced spin moments in Figure 2e might also be related to the random orientation of the particles. Most likely, the deposition process also leads to statistically distributed magnetic anisotropy axes. The competition between the anisotropy energy and the exchange interaction with the substrate can then lead to non-collinear spin structures in the particles [66]. As a result, the magnetic spin moments of the particles are no longer parallel to the magnetization of the substrate but are canted by a certain angle towards the direction of the individual anisotropy axis as sketched in Figure 2g. Thus, in the present XMCD experiments we would only probe the averaged projection of the magnetization in the particle ensemble which leads to an apparently reduced magnetic moment in the sum rule analysis. The fact that we observed bulk-like spin moments in similar Fe nanoparticles on hcp Co(0001)/W(110) [38] may reflect that the exchange interaction clearly dominated over the magnetic anisotropy energies in the latter system while on fcc Ni(111)/W(110) the anisotropy energy strongly determines the magnetization direction of the particles [66]. Alternatively, a substrate-induced fcc/bcc competition in the nanoparticles could lead to reduced spin moments in Figure 2e as, e.g., observed for Fe nanostructures grown on fcc Cu(111) in [67].

## Conclusion

We have presented a combined XMCD, RHEED, and STM study on the magnetic, structural and morphological properties of Fe nanoparticles deposited from the gas phase onto single crystalline substrates under soft landing conditions. In the XMCD experiments strongly enhanced magnetic orbital, but reduced magnetic spin moments (compared to respective bulk values) in Fe nanoparticles on fcc Ni(111)/W(110) were found. In situ RHEED revealed a random orientation and bulk-like lattice constants in the Fe nanoparticles, and thus excludes strain effects as the main origin of the altered magnetic moments. Instead, we propose the presence of deposition-induced dislocations in the Fe nanoparticles as the main contribution for the enhanced magnetic orbital moments. The reduced spin moments could be due to the competition between randomly oriented anisotropy axes and the exchange interaction with the substrate. Furthermore, we have shown that below a critical size – being 4 nm in the case of Fe on W(110) – the particles are able to spontaneously align on a single crystalline substrate. STM experiments, however, hint at a complex reshaping of the particles which may happen simultaneously.

The experimental results demonstrate the impact of the deposition kinetics on the physical properties of supported nanoparticles, even under soft landing conditions. Combining RHEED and STM with other methods provides a lot of information on the nanoparticles. The complex relation of structure, orientation and morphology also underlines the need for experiments with single particle sensitivity [68]. Such data are not only relevant for magnetism, but also for charge transport phenomena and catalytic activities of supported nanoparticles in heterogeneous catalysis. Our findings are also relevant for chemically produced particles which are subsequently dispersed on a surface. In such a case, the dispersion process will also result in a random orientation of the particles with the respective consequences for their properties. Rapid annealing by short laser pulses may provide a tool to obtain uniformly orientated particles in this situation, but the shapes can be affected in an undesired manner. Thus, to gain full control over shape and orientation – even in mono-dispersed nanoparticle deposits that are potentially of interest for applications as well as for fundamental research – remains challenging.
